# Evaluation of Patient Experiences Regarding Pharmacist-Administrated Vaccination and Attitude towards Future Additional Pharmacy Services in Poland

**DOI:** 10.3390/vaccines10091479

**Published:** 2022-09-06

**Authors:** Izabela Grzegorczyk-Karolak, Aneta Zglińska-Pietrzak, Izabela Weremczuk-Jeżyna, Sylwia Kałucka

**Affiliations:** 1 Department of Biology and Pharmaceutical Botany, Medical University of Lodz, 90-151 Lodz, Poland; 2 Department of Mathematics, Medical University of Lodz, 90-151 Lodz, Poland; 3 Department of Coordinated Care, Medical University of Lodz, 90-251 Lodz, Poland

**Keywords:** COVID-19 vaccination, health care workers, motivation, pharmaceutical service, patient, pharmacist, pharmacy, satisfaction, vaccine

## Abstract

In order to increase the number of vaccinations performed during the COVID-19 pandemic in Poland, a significant change was introduced in the legislation allowing for the vaccination to also be performed in pharmacies. A cross-sectional survey was conducted among those who chose a pharmacy as a vaccination point during the pandemic COVID-19. The aim of the study was to determine the overall level of patient satisfaction with pharmacist-administered vaccination in pharmacies and to examine patient opinions regarding the further expansion of pharmacy services. A patient survey was conducted immediately after vaccination in the period from January to March 2022. A total of 398 questionnaires were completed. The respondents reported high satisfaction with pharmacist-administered vaccinations (94.5%). In addition, the majority of consumers felt safe during the vaccination procedure (98.5%), and 88.4% declared they would come for other vaccinations at the pharmacy. The two main reasons for choosing this vaccination place were easy access regarding location (94.2%) and proposed vaccination hours (95.2%). The participants reported high levels of satisfaction concerning the pre-vaccination interview (91.2%), information level before immunization (91.5%), injection technique (96.7%), adjusting the premises to the service (95%) and general care in pharmacy after with vaccination (87.7%). The majority of respondents supported the future expansion of pharmacist-administered services, although the support rate ranged from 52% to 83% depending on the type of service offered. The majority of patients supported the continuation of ongoing prescriptions, blood pressure and blood glucose measurement services by pharmacists. Our findings indicate that the involvement of pharmacies in vaccination programs and other public health services met high levels of patient acceptance.

## 1. Introduction

Mass immunization has resulted in a reduction in the health burden of polio, varicella, measles or rubella, and the global elimination of some infectious diseases, such as smallpox [1]. The recent COVID-19 pandemic also confirmed the effectiveness and importance of vaccination as a preventive agent against infectious diseases, for both the individual and the global population. However, the effective implementation of vaccination programs in societies remains an important challenge [2]. Such vaccination programs can limit the spread of the virus and its further mutations. Achieving high vaccination coverage is determined not only by the universal availability of vaccines, but also by the ease of access to vaccination sites and the number of health care workers. To date, the vaccination system in Poland required two arranged visits to the GP during working hours, only on weekdays. This is often a significant barrier for patients in the case of vaccination against SARS-CoV2 or influenza, which requires regular boosters [3].

Pharmacist-administered vaccination services have been available in various countries for several years, with the first being initiated in the United States of America in 1996 [4]. Since then, the possibility has also extended to other countries including Portugal, New Zealand, Canada, the United Kingdom and Australia [5], and legislation is being prepared in several others [6]. Such changes in the vaccination system were also forced by the COVID-19 pandemic in Poland.

In Poland, changes in legislation were adopted in April 2021 to allow the expansion of pharmaceutical services to include vaccines against COVID-19 [7]. Since July 2021, Polish pharmacists have been allowed to vaccinate healthy adults after successfully completing theoretical and practical training and acquiring a relevant certificate of competence for vaccination. The regulations also specify that pharmacies in which vaccination takes place must have a private area for the service, as well as sufficient space for the pre-vaccination interview and for the recipient to remain under observation following the vaccination [8]. Medical Centre of Postgraduate Education data indicates that as of November 2021, 8363 of the 36,527 registered pharmacists in Poland have completed training in vaccination against COVID-19 [9]. About 20% of trained pharmacists have participated in vaccinations, and from July 2021 to March 2022, over 1.8 million individuals have been vaccinated in pharmacies, this accounts for 7–8% of the total administered doses of the vaccine against COVID-19 at that time in Poland [10]. However, a survey performed between February and August 2020, i.e., a year earlier, found that 88% of surveyed pharmacists, who had no contact with vaccination at the pharmacy, believed that pharmacies were not suitable to provide vaccination services [11]. The pharmacists also reported concerns about significant increases in workload and insufficient training courses.

Before further medical services provided by pharmacists can be introduced permanently, it is important to determine the overall level of patient satisfaction with the new pharmacy services. The main objective of this study was to determine the level of patient satisfaction with pharmacist-administered COVID-19 vaccination at pharmacies in Poland and identify the factors associated with this choice of vaccination location. The study also examines patient opinions regarding the inclusion of additional services at pharmacies. The analysis examines the influence of gender, age, place of residence, education level and doses taken of COVID-19 vaccines on the responses. This is the first such study on this topic to be performed in Poland.

## 2. Materials and Methods

### 2.1. The Study Questionnaire

A primary version of the survey was created based on an analysis of the most frequently reported issues and questionnaires used in other studies [12,13,14]. The primary version of the questionnaire was validated by 14 individuals including three academic staff, three pharmacists working in the pharmacy, and eight patients), and the feedback was used to correct the questionnaire before implementation. At the request of the patients and pharmacists participating in the validation study, the responses were simplified: the questions had to be simple and clear to all participants. The survey should not take longer than 10 to 15 min to complete, as this is the recommended post–vaccination observation time, when the patient is at the pharmacy.

Ultimately, the cross-sectional questionnaire was divided into five sections with a total of 23 questions, to gather basic demographic and general information (i), reasons for vaccination at the pharmacy (ii), prior experiences regarding the different aspects of the pharmacist vaccination service (iii), satisfaction with regarding the pharmacist vaccination service (iv), and opinions on further expansion of the services in the pharmacies (v). Most answers used a 3-point scale: ‘agree’, ‘disagree’ and ‘I have no opinion’. When an answer in question 16 was missing, it was denoted as ‘no answer’. The final version of the study questionnaire is provided in the Supplementary Materials (Supplementary Table S1). The sample size was calculated by the Raosoft sample size calculator [15]. Based on the estimated number of doses of the COVID-19 vaccines administered in Poland in pharmacies so far and a response distribution of 50%, the required sample size was 385 for a confidence level of 95% and a 5% margin of error. In the study, a total of 398 questionnaires were completed.

### 2.2. The Study Design

The questionnaire, together with an informative letter, were provided to the pharmacy managers/proprietors in December 2021 and January 2022 to obtain approval for including the pharmacy and pharmacists in the study. The criterion for including a pharmacy in the study was that it should perform vaccinations at least three days a week, as this would make it possible to ensure a representative population of vaccinated individuals. If the level of immunization in the pharmacy was low, there was a concern that the only the family and friends of the pharmacy employees were receiving vaccinations, which would disturb the objectivity of the study. Therefore, if the number of patients for vaccination decreased significantly and the number of completed questionnaires was low at any vaccination point, it was withdrawn from the study. This manuscript includes data collected at pharmacies in Poland between January and March 2022: when the vaccination coverage level had slightly decreased due to the end of the fourth COVID-19 wave, which made it possible to involve pharmacists in the giving out of questionnaires. Ultimately, data were collected in eight pharmacies, both in large and smaller cities. In participating pharmacies, all vaccinated patients were invited to complete a questionnaire. Information was attached to each questionnaire about the aims of the study. Patients were also advised about the confidentiality of the study: the survey did not collect any information related to their identity or to the pharmacy. Participation in the survey was voluntary and anonymous. The participants were informed by the pharmacist that the return of a completed questionnaire indicated consent to its use.

### 2.3. Statistical Analysis

Demographic characteristics were summarised using means and standard errors or counts and proportions. In the analyses, the answers were stratified by sex, levels of education, place of residence and age group (G1: ≤35 years, G2: between 36–50 and G3: ≥ 51 years). As the answer ‘yes’ was the most relevant, for statistical analysis, the questions that had three options combined the answers ‘no’ and ‘I have no opinion’ into one option. The Chi-square test or, depending on the assumptions met, tests from this family (V-square, Chi-square with Yates correction) were used to test the relationship between responses and demographics. The significance level was set at 5%. When the relationship was found to be significant, odds ratios and 95% confidence intervals (Cl) were also calculated and reported. Statistical analysis was carried out using Statistica Software version 13.3 (StatSoft, Krakow, Poland).

## 3. Results

### 3.1. Demographic Characteristic

Table 1 shows the demographic data of the respondent population. The majority of consumers were female (56.8%) and the mean age of the respondents was 40.3 ± 14.8, range 16 to 81 years. The number of completed questionnaires received from respondents was 205 from large cities (51.5%), 143 from small towns (35.9%), and only 49 (12.3%) from villages. The respondents reside in nine out of the 16 voivodships (units of administrative division) in Poland, with most coming from the Pomorskie and Lodzkie voivodeships, where most pharmacies participating in the study were located. 

The majority of consumers had a higher level of education (i.e., a Bachelor’s or Master’s degree) or had completed secondary school (both 40.7% each); in addition, 12.5% had completed secondary vocational education, and only 6% primary education (Table 1). Most of the respondents were vaccinated at the pharmacy with the Pfizer/BioNTech vaccine (98%). Only isolated cases were vaccinated with other vaccines during the study period: 1.8% with the Moderna vaccine and 0.25% with Johnson & Johnson vaccine. Most of the patients had the third dose of the COVID-19 vaccine (77.4%) at the pharmacy. Also, 15.1% of the patients were vaccinated at the pharmacy for the second dose, and 7.5% for the first dose.

### 3.2. Reasons for Vaccination

The reasons for choosing to be vaccinated in a pharmacy are visualized in Table 2. Most indicated a convenient location (375 respondents; 94.2%) and convenient opening hours (379 respondents; 95.2%). In addition, 74.9% of respondents reported good previous experiences with pharmacy services. Almost half (43.2%) chose a pharmacy because it was the only vaccination site available to date. In addition, 7.8% had no knowledge of the places of vaccination: they signed up for vaccination directly at the pharmacy and they did not have any information about the availability of vaccinations in other locations. Furthermore, 13.6% of participants were not aware of the availability of vaccination at their GP, suggesting that they rarely visit the outpatient clinic. However, as the survey was performed after the peak of the vaccination boom, over 60% of people reported that they had no problem accessing their outpatient clinic.

Statistical examination according to sex, age, place of residence, level of education, and vaccine dose are provided in Supplementary Table S2. A higher level of education was associated with a lower tendency for the participant to indicate they chose the pharmacy because it was the only place available at that time. Significantly more older people (especially those over 50 years old) than younger people reported choosing the pharmacy because the vaccine could be obtained quickly (*p* = 0.001) and having a previous positive experience with pharmacy services (*p* = 0.002). In contrast, the youngest age group were more likely to indicate easy access and a favorable location as reasons for using the pharmacy (*p* = 0.008). 

Those who were vaccinated for the third time against COVID-19 were more likely to indicate convenient opening hours and better accessibility as reasons compared to those being vaccinated for the first or second time (respectively, *p* = 0.017 and *p* = 0.009). Easy accessibility was also more important for people from small cities than the other locations, especially those living in the countryside. The respondents from the small cities (less than 100,000 inhabitants) were significantly more likely to indicate good experiences with pharmacist services as an important reason for choosing a pharmacy vaccination (Supplementary Materials Table S2).

### 3.3. Experiences with Vaccination Service at a Pharmacy

Almost half of the respondents registered for vaccination at the pharmacy in person or by telephone contact (44.2%). Slightly fewer people used the special government website for registration (41%), and only 14.8% used the 24-h nationwide hotline for COVID-19 vaccination.

People from the youngest age group, those with higher education, and those living in cities with more than 100,000 inhabitants, most often chose the form of electronic registration for vaccination using an internet site (Supplementary Materials Table S3). In contrast, the oldest age group, people from smaller towns and villages as well as people with only primary and secondary vocational education, preferred personal or telephone registration at a selected pharmacy. No statistically significant differences in form of registration were observed according to sex or vaccine dose. 

Opinions were also gathered regarding the interview prior to vaccination, information received from a pharmacist prior to vaccination, vaccination location organization, vaccination technique, and post-vaccination care. The responses are summarized in Table 3.

Over 90% of the respondents positively assessed both the pre-vaccination interview conducted by the pharmacist and the information they obtained about the procedure (Table 3). Almost all respondents (96.7%) were satisfied with the vaccination technique, and none of the single people expressed a negative opinion. Most participants were satisfied with the organization of the location (95%), and only two people expressed a negative opinion on this issue. Most of those vaccinated at the pharmacy also had a positive opinion of post-vaccine care. However, over 12% of the subjects chose the answer ‘I do not know’, which could result from the fact that a significant percentage of consumers did not stay in the pharmacy for the recommended 15 min after the vaccination, especially among those vaccinated against COVID-19 for the third time.

Respondents vaccinated against COVID-19 for the third time were more positive about the pre-vaccination interview collected in the pharmacy (*p* = 0.0001) and the conditions of the premises that were created at the pharmacy for vaccination (*p* = 0.0002) compared to those vaccinated for the first or second time; this may be due to their greater experience in this area (Supplementary Materials Table S4). Patients vaccinated more than once were also more positive about the technique of vaccination performed by pharmacists (*p* = 0.0001).

Some differences were also observed with regard to age group and place of residence. The youngest customers and those living in cities, assessed the injection technique significantly more positively than older or rural respondents (Supplementary Materials Table S4). In addition, women were significantly more likely to positively assess the information obtained before vaccination than men. Finally, the youngest age group, inhabitants of large cities and people with higher or primary education expressed significantly better opinions regarding post-vaccination care (Supplementary Materials Table S4).

### 3.4. Summary of Pharmacist-Administrated Vaccination

As shown in Table 4, 90% the study participants agreed that the pharmacists are competent to administer the vaccine. Of the respondents, 392 (98.5%) felt safe and comfortable during the vaccination procedure at the pharmacy, and only slightly fewer (94.5%) were satisfied that they decided to vaccinate against COVID-19 at that location. None of the respondents answered “no” to these questions. In addition, 88.4% of respondents expressed a wish to revaccinate at the pharmacy in the future.

No statistically significant differences in the sense of safety over time in the pharmacy vaccination and satisfaction with using the pharmacy for vaccination was noted between the different groups of respondents (Supplementary Materials Table S5). On the other hand, the competencies of pharmacists were rated significantly higher by women and respondents under the age of 35. Moreover, people with higher education were more likely to declare coming to the pharmacy for further vaccinations than other groups, especially those with primary education (*p* = 0.034).

### 3.5. Attitude to the Expansion of Pharmacy Services

The objective of the survey was also to provide data to support discussions for the commissioning of other services in pharmacies in Poland. Most respondents were open to other pharmaceutical care services (Table 5).

The greatest number of respondents (more than 80%) supported blood pressure and blood glucose measurement services and the continuation of ongoing prescriptions for drugs used on a permanent basis by pharmacists (Table 5). The respondents also supported training sessions on the operation of medical equipment such as insulin pens, glucometers, and inhalers (72.4%). Some participants (65–70%) would like also to have the possibility to consult on different health-related issues with pharmacists such as weight control, diet, or therapy monitoring with new drugs. The smallest group, but still more than half of those taking part in the study, supported the participation of pharmacists in quitting smoking.

No significant differences were observed between individual groups of respondents regarding the extension of pharmaceutical services to blood pressure measurement, blood glucose measurement, or support in quitting smoking (Supplementary Materials, Table S6). Women supported the service of training sessions on using glucose meters, inhalers, and other medical equipment significantly more than men (*p* = 0.007). Subjects in the G2 age group (between the ages of 36 and 50) favor the introduction of dietary advice to pharmaceutical services more often than the other age groups. On the other hand, people living in large cities were more in favor of continuing ongoing prescriptions for drugs than residents of towns and villages (*p* = 0.015) (Supplementary Materials Table S6). People with primary education were more likely to support the performance of weight measurement and BMI calculation at a pharmacy (*p* = 0.026) as well as monitoring of therapy with new drugs (*p* = 0.039). Respondents vaccinated against COVID-19 several times demonstrated the greatest support for expanding dietary counseling by pharmacists (*p* = 0.011).

## 4. Discussion

Pharmacists are healthcare professionals whose qualifications are currently not fully utilized by the health care system. In some countries, in addition to dispensing medications, preparing them, and advising patients, pharmacists play an important role in healthcare by actively participating in interdisciplinary patient treatment. Recent changes in legislation may result in similar changes in Poland. 

One year since the emergence of the COVID-19 pandemic, rapid response has been implemented to control the spread of the virus. In order to maximize the public health benefit of available vaccines, the competencies of pharmacists were expanded and the possibility of vaccination was introduced in generally-accessible pharmacies in Poland. Appropriate legislative changes were also introduced during the COVID-19 pandemic in several other countries. Pharmacy vaccination services complement those provided by general practitioners and can help improve overall coverage and the vaccination rates for patients. In Europe, vaccinations are currently performed by pharmacists in Portugal, Ireland, Great Britain, France, Switzerland, Denmark, Norway, Greece, Germany, and Italy [16]. 

In most countries, pharmacist-administrated vaccinations were rolled out on a phased basis, starting with pilot programs. In contrast, the Polish vaccination strategy has changed rapidly to support the vaccination provision during the ongoing global pandemic. However, it was reported that patients expressed a positive attitude toward the implementation of vaccination services in pharmacies before the changes in vaccination policy took effect [17] 

In our present study, the participants evaluated the level of service following vaccination and noted their reason for being vaccinated in a pharmacy; they also indicated whether they would support expanding the range of services offered by the pharmacy. This study, the first such paper since pharmacist-administered vaccination began in Poland, found that overall consumer satisfaction was 94.5%. Consumers were satisfied with the interview and information provided before vaccination, the skills, and professionalism of the pharmacists, and post-vaccination care. Most respondents declared that they could receive further vaccination at the pharmacy in the future. 

This finding is similar to those reported in other countries; overall satisfaction with pharmacist-administered vaccination services internationally was 97–99% [13,14,18]. Papastergiou et al. [18] report a high degree of patient satisfaction in Canada, with 92% of patients indicating they were very satisfied with the pharmacist’s injection technique and the services they received, and 99% would recommend the service. In Australia, 99% of 434 customers were satisfied with the service overall and 97% declared an intention to get community pharmacist-administered vaccinations in the future [5]. Similarly, most respondents in New Zealand reported that pharmacy vaccination was carried out in a safe and professional manner, and more than 80% of the respondents declared they would definitely be immunised by a pharmacist again [14]; in addition, 93% of respondents were also satisfied with the quality of the explanation regarding immunization provided at pharmacies.

In the present study, the most important reasons given for choosing a pharmacy as a vaccination site was a convenient location and convenient opening hours. This suggests that the pharmacy customers expected the procedure to be and easily accessible and quick. Similarly, previous studies also indicated that easy access, suitable opening hours, and convenience were important reasons for choosing a pharmacy [19,20,21,22]. Convenience and accessibility seem to be particularly attractive reasons for pharmacist-administered vaccination, especially for busy people [19,20]. Indeed, as pharmacies could be open seven days a week, with longer opening hours than most medical outpatient clinics, they may represent a more attractive option for vaccination than a GP surgery. In pharmacies, the peak times for vaccination in England and America were mid-morning and after 6 pm, so out of working hours of the GP [23]. Similarly, patient satisfaction with the pharmacy-based service during a pilot program on the Isle of Wight was high, with accessibility seen as a major advantage over general medical practice [19]; none of the earlier vaccination programs had such a positive effect. Patient responses were positive towards the service, with 98% indicating that they would use the pharmacy service again.

The accessibility of pharmacies with long opening hours, and the lack of any need to make appointments, largely favours the patients and can positively influence their decisions to get vaccinated in pharmacies [24]. In other studies, the lack of any requirement for an appointment was seen as a significant factor in choosing pharmacy vaccination [25]. While a prior appointment was required in the case of COVID-19 vaccinations in Poland, due to the great interest, it was often possible to obtain vaccinations without an appointment if free doses of the vaccine were available.

A UK study found that pharmacies appear to cater to those who find it less easy to access GP surgeries [26]. Indeed, Kowalczuk et al. [17] report that 80% of respondents found pharmacies significantly more accessible than outpatient clinics. The awareness that pharmacy vaccination takes less time increased with the level of education. Similarly, most of the respondents (60.0%) agreed that pharmacist-administered vaccination would relieve the burden on doctors and nurses, and this awareness increased with the level of education. On the other hand, 60% of our study participants declared that they did not have problems with access to their GPs. Similarly, in a Swiss study, most respondents (75.7%) stated that the lack of availability of a GP did not increase the likelihood of using a pharmacy for vaccination [25]. These results suggest that part of customers actively chose pharmacies as an alternative option rather than being forced to do it.

As observed in the COVID-19 pandemic, a potential benefit of pharmacist-administered immunization would be to free GPs and hospitals to concentrate resources on treating those already sick. It may also help overcome obstacles to vaccine access. It is hence in line with a key goal of the World Health Organisation (WHO), i.e., increasing the infectious disease vaccination rate in the population to reduce cases and healthcare costs [27].

In the USA, allowing pharmacists to vaccinate was associated with higher influenza vaccination rates for individuals aged 65 years and older [28]. Influenza vaccinations delivered by pharmacists in the USA between 2007 and 2013, increased from 3.2 million to 20.9 million [29]. According to some data, administration of the flu vaccine by pharmacists improved vaccination rates among patients, especially occasional vaccine recipients, and patients who may not otherwise have had the opportunity. In Poland, it is too early to carry out such analyses, because it is the first year that pharmacists have been authorized to vaccinate. Meanwhile nearly one-third of respondents in Canada and a quarter in the USA indicated that they would not have been vaccinated this year if pharmacist-administered vaccination were not available [18,30]. In the 2015/16 flu season, flu vaccinations were commissioned for the first time in pharmacies in some areas in England, and since then, in this location, higher vaccination coverage has been reported [26]. Also, many respondents in Switzerland (40.9%) received the seasonal influenza vaccination for the first time in a pharmacy, which clearly suggest that expanding the role of pharmacists improves overall access to vaccinations [25]. Similarly, the main reason given by respondents from New Zealand for not being previously immunised was that they were ‘too busy’ (39%) and only the easy availability of vaccinations administered by pharmacists encouraged them to do so [14]. There is an expectation by many consumers that health services should be readily accessible, and restriction of availability negatively affects the willingness to use them.

Due to the success of pharmacist-administrated vaccination, some countries permit the pharmacist to administer vaccinations against other diseases; for example, pharmacies have been administering influenza, hepatitis A and B, and dTP vaccines in the USA [31,32,33], dTP vaccines in New Zealand, and MMRV and hepatitis B vaccines in the UK [5,26]. Moreover, many pharmacy schools in the USA have long been providing regular vaccination training [21].

While some research suggests that pharmacy flu vaccination services are predominantly used by people with higher education [23], other studies, including the present one, indicate that the service was accessed by patients from a broad spectrum of educational backgrounds [26]. Nevertheless, in the present study, the respondents with higher education were significantly more likely to report that they would come to the pharmacy for the next vaccination. Moreover, in our study, people over 50 years old and respondents from small cities significantly more often declared that trust in pharmacists and good experiences with pharmacist services were for them an important reason for choosing pharmacy vaccination. This is easily explained by the greater frequency of visits to the pharmacy and better knowledge of individual pharmacists by older people than younger people. Also, closer contact between staff of nearby pharmacy and residents in a small community are observed compared to larger ones; in the latter case, there are more pharmacies, but with fewer regular customers. In addition, rural areas tend not to have pharmacies; as such, for rural residents, there is no clear difference in accessibility between a pharmacy and a health centre. It is difficult to establish contact between pharmacists and customers when the pharmacy is a long distance away from the place of residence, as can be in the case when the pharmacy is located in the home town.

During the COVID-19 pandemic, the competencies of pharmacists in Poland were expanded to include not only vaccination, but also the provision of pharmaceutical care services, such as medication reviews or issuing pharmaceutical prescriptions [34]. While the vaccinations have already been implemented, the development of pharmaceutical care in Poland requires further appropriate legislation. It also seems necessary to introduce training programs and modify curricula to meet expectations in terms of pharmacist competencies. Also, in a survey conducted in pharmacies in Poland in 2020, the employees reported that practical training courses were insufficient [11]. In a study carried out in Hungary, the majority of study participants advocated including pharmaceutical care, clinical therapeutics and clinical pharmacy competencies in curricula to adapt to advances in Pharmacy and provide adequate preparation for expanding skills and competencies among pharmacists [35]. The study reflects global trends recommending changes focusing on everyday pharmacy practice in the education system [36,37].

According to a recent study, pharmacists in Poland have expressed their readiness to expand the scope of services they offer [38]; therefore, the aim of our study was also to analyse the perception of patients regarding the expansion. Expanding the role of pharmacists to include vaccinations and other services could have a potentially positive impact on public health. Pharmacists are highly-trained health professionals who can complement medical services provided by doctors and thus improve access to them. Such services are being increasingly offered in pharmacies in some countries, and these are in line with the concept of enhancing pharmacists’ competences, as promoted by WHO and the International Pharmaceutical Federation [39]. The possibilities for including pharmacists more actively in the health care system are broad: participation could include services such as drug reviews including identification of drug interactions and pharmacovigilance, maximization of treatment through preventing, identifying, and solving medication-related problems, and prevention programs regarding healthy eating or stopping smoking. Consumers frequently visit pharmacies, allowing pharmacists to target many customers, especially those who may not see a GP regularly [40].

Our respondents are generally supportive of pharmacist service expansion, especially with regard to measuring blood pressure, blood glucose, or the continuation of ongoing prescriptions for drugs used on a permanent basis. Such activities can support the early diagnosis of some diseases, e.g., hypertension and diabetes. Patients are also open to educational services regarding pharmaceutical care. They would also be willing to take part in training sessions on the operation of medical equipment. Being able to use such equipment as insulin pens and glucometers can be very helpful in regulating blood glucose levels among diabetics. A similar willingness has also been noted in other studies, particularly concerning the role of pharmacists about healthy eating and recovery from addiction [41]. Additionally, a recent systematic review investigating pharmacist-delivered interventions found that those concerning smoking cessation were effective and that pharmacies were a feasible option for weight management interventions [40]. Patients would like also to have the possibility to consult pharmacists about medication and health-related issues; indeed, the vast majority of respondents have been found to have a positive opinion about the pharmacy staff [41]. A large proportion of our respondents also reported trusting pharmacists, which is a great starting point for expanding pharmaceutical services. However, the introduction of such services in Polish pharmacies requires further legislation, including the standardization of patient service requirements or reimbursement for pharmaceutical care. In addition, there is a need for programs aimed at bringing pharmaceutical care closer to the general community in Poland. In our survey, 6–11% of the respondents did not provide any answers to questions relating to a particular service; this may be related to the lack of knowledge of what such service consists of or how it could be run in a pharmacy.

## 5. Limitations

Our study has several limitations. Inclusion was voluntary for both pharmacies and respondents, which can lead to selection bias. The respondents seem to generally favour vaccinations, because the vaccination itself was voluntary. Moreover, the respondents had already chosen to be vaccinated in a pharmacy and wanted to take the time to complete the survey; as such, they were likely to have a positive or at least indifferent attitude to the competencies and knowledge of pharmacists. However, people who are particularly dissatisfied tend to express their opinions and the possibility of submitting a complaint, and would probably not refuse to participate in the survey.

In addition, pharmacies participating in the study covered only part of the country (they were located mainly in 2 out of 16 voivodships); as such, there may be some limitations on generalisability. Additionally, it was not possible to monitor the total number of vaccinations performed in the pharmacies; therefore, conclusions on rates and selection should be drawn tentatively.

The questions relating to pharmaceutical care issues are a pilot study of patient expectations of future additional services in pharmacies. This part of the survey did not include a detailed analysis of the needs of individual respondents. It is possible that some of the respondents who answered ‘no’ to individual inquiries in the field of pharmaceutical care do not oppose the use of such services in pharmacies *per se*, but do not see any need for themselves; for example, they do not smoke or suffer from diabetes, they do not need to be on a diet or they use their own blood pressure monitor at home.

## 6. Conclusions

Pharmacists are highly-trained and easily-accessible health professionals who can increase not only vaccination uptake in the community, but also provide other health care services. Such services are being increasingly offered in other countries, including in Poland, following recent changes in legislation. Pharmacy vaccination services complement those provided by GPs to help improve overall coverage and vaccination rates for patients. These services seem to be particularly attractive to those of working age, most likely due to the convenience and accessibility of pharmacies. This service received a very positive evaluation from our participants and revealed positive consumer experiences. In addition, the respondents declared positive attitudes toward other services that pharmacies could provide, and which would support a burdened healthcare system. However, there is little general awareness of the range of potential health promotion services in Poland, and campaigns are needed to provide information about such possibilities and the purposes of providing services in pharmacies.

## Figures and Tables

**Table 1:** Demographic characteristic of participants.

Group Characteristic	
Sex	
Female	226 (56.8%)
Male	172 (43.2%)
Age, mean ± SD	40.3 ± 14.8
Voivodeship	
Lodzkie	252 (63.3%)
Pomorskie	113 (28.4%)
Others	33 (8.3%)
Place of Residence	
Rural	49 (12.3%)
city of less than 100,000 inhabitants	143 (35.9%)
city above 100,000 inhabitants	206 (51,8%)
Education	
Primary	24 (6.0%)
Secondary vocational	50 (12.6%)
Secondary general	162 (40.7%)
Higher	162 (40.7%)
COVID-19 vaccine type	
Pfizer-BioNTech	390 (98.0%)
AstraZeneca	0 (0.0%)
Moderna	7 (1.8%)
Johnson *&* Johnson	1 (0.25%)
Vaccine dose	
first	30 (7.5%)
second	60 (15.1%)
third	308 (77.4%)

**Table 2:** The reasons for getting vaccinated against COVID-19 at the pharmacy.

Reason	Yes	No	I Do Not Know
The pharmacy was the only vaccination site available	172 (43%)	195 (49%)	31 (7.8%)
Problems with access to my clinic	100 (25.1%)	244 (61.3%)	54 (13.6%)
Convenient opening hours of the pharmacy	279 (95.2%)	1 (0.3%)	18 (4.5%)
Convenient location	375 (94.2%)	3 (0.8%)	20 (5%)
Good experiences with the pharmacy service	298 (74.9%)	4 (1.0%)	96 (24.1%)

**Table 3:** Opinions regarding experiences of immunization against COVID-19 at the pharmacy.

Positive Opinion on	Yes	No	I Do Not Know
the interview prior to vaccination	363 (91.2%)	0 (0.0%)	35 (8.8%)
the information received prior to vaccination	364 (91.5%)	0 (0.0%)	34 (8.5%)
the technique of vaccination	385 (96.7%)	0 (0.0%)	13 (3.3%)
the premises organized for vaccination	378 (95%)	2 (0.5%)	18 (4.5%)
the post-vaccination care	349 (87.7%)	41(0.3%)	48 (12.1%)

**Table 4:** Opinions regarding satisfaction with immunization at the pharmacy.

Final Opinion	Yes	No	I Do Not Know
I felt safe during the vaccination procedure	392 (98.5%)	0 (0.0%)	6 (1.5%)
Pharmacists are competent to vaccinate	359 (90.2%)	0 (0.0%)	39 (9.8%)
I am glad that I got vaccinated at the pharmacy	376 (94.5%)	0 (0.0%)	22 (5.5%)
A wish to revaccinate at the pharmacy in the future	352 (88.4%)	1 (0.3%)	45 (11.3%)

**Table 5:** Attitude of respondents to the expansion of different pharmacy services.

Pharmacy Service	Yes	No	No Answer
Blood pressure control	323 (81.1%)	50 (12.6%)	25 (6.3%)
Glucose control	322 (80.9%)	50 (12.6%)	26 (6.5%)
Measuring the weight and height and BMI calculation	281 (70.6%)	90 (22.6%)	27 (6.8%)
Advice about specialized diets	276 (69.3%)	93 (23.4%)	29 (7.3%)
Use a glucometer, pen for insulin injection or inhaler	288 (72.4%)	74 (18.6%)	36 (9.0%)
Supporting of the process of quitting smoking	206 (51.8%)	148 (37.2%)	44 (11.0%)
Continuation of ongoing prescriptions	330 (82.9%)	38 (9.6%)	30 (7.5%)
Monitoring of the therapy with newly introduced drugs	261 (65.6%)	97 (24.4%)	40 (10.0%)

## Data Availability

The dataset we analyzed for the current study is available from the corresponding author on request.

## Supplementary Materials

The following supporting information can be downloaded at: https://www.mdpi.com/article/10.3390/vaccines10091479/s1, Table S1: The survey questionnaire; Table S2: The reasons for getting vaccinated against COVID-19 at a pharmacy in relation to basic characteristics of participants; Table S3: Method of registration for vaccination at pharmacies in relation to basic characteristics of participants; Table S4: Opinions regarding experiences of immunization against COVID-19 at the pharmacy in relation to basic characteristics of participants; Table S5: Opinions regarding satisfaction with immunization at the pharmacy in relation to basic characteristics of participants; Table S6: Attitude to the expansion of pharmacy services in relation to basic characteristics of participants.
