# Supplementary material for: Evaluation of Patient Experiences Regarding Pharmacist-Administrated Vaccination and Attitude towards Future Additional Pharmacy Services in Poland
Source: Vaccines (Basel). 2022 Sep 6;10(9):1479. doi: 10.3390/vaccines10091479 (PMC9501353; doi:10.3390/vaccines10091479)
Supplement: Supplementary file 1 [file vaccines-10-01479-s001.zip › vaccines-1856615-supplementary.pdf]

Table S1. The survey questionnaire

**Questions**	**Answers**
Why did you decided to get vaccinated at the pharmacy	
1. The pharmacy was the only vaccination site available to me when I wanted to	• Yes<br>• No<br>• I have no opinion
2. I have problems with access to my GP	• Yes<br>• No<br>• I have no opinion
3. The opening hours of the pharmacy are fine for me	• Yes<br>• No<br>• I have no opinion
4. The pharmacy is easily accessible to me (e.g. proximity to home / work, parking, public transport connection)	• Yes<br>• No<br>• I have no opinion
5. I have already good experiences with the pharmacy service and I trust the pharmacists	• Yes<br>• No<br>• I have no opinion
Different vaccination aspects	
6. How did you arrange the vaccination at the pharmacy:	• I registered electronically via e-registration, patient gov.pl<br>• I used the 24/7 helpline 989<br>• in person during a visit to the pharmacy or phone
7. I am satisfied with the interview before the COVID-19 vaccination at the pharmacy.	• Yes<br>• No<br>• I have no opinion
8. I am satisfied with the information I have received from the pharmacy prior to the vaccination	• Yes<br>• No<br>• I have no opinion
9. I am satisfied with the technique of vaccination in the pharmacy	• Yes<br>• No

	• I have no opinion
10. I am satisfied with the premises that have been organized in the pharmacy for vaccination	• Yes<br>• No<br>• I have no opinion
11. I am satisfied with the post-vaccination care in the pharmacy	• Yes<br>• No<br>• I have no opinion
Summary of pharmacist-administrated vaccination	
12. I felt safe throughout the vaccination procedure at the pharmacy	• Yes<br>• No<br>• I have no opinion
13. I believe pharmacists are competent to administer the vaccine	• Yes<br>• No<br>• I have no opinion
14. I am glad that I got vaccinated against COVID at the pharmacy	• Yes<br>• No<br>• I have no opinion
15. I would come to the pharmacy for another vaccination (not only against COVID-19)	• Yes<br>• No<br>• I have no opinion
Pharmaceutical care	
16. What other services do you want to be available at the pharmacy?<br><br>1. Ability to control blood pressure<br>2. Ability to control glucose levels<br>3. Ability to measure weight and height and calculate BMI	• Yes<br>• No

4. Possibility of advice from a pharmacist about specialized diets, e.g. slimming diets, lowering cholesterol, diabetic diets, diets for stomach diseases, etc.<br>5. Training sessions on the operation of medical equipment such as insulin pens, glucometers and inhalers<br>6. Advice and control of quitting smoking<br>7. Possibility to continue ongoing prescriptions by pharmacists<br>8. Monitoring of the course of therapy (well-being, occurrence of side effects) with newly introduced drugs	
Demographic data	
17. Age	in years ………………………………
18. Sex	• Woman<br>• Man
19. Voivodeship	in years ………………………………
20. Place of residence	• City with more than 100,000 inhabitants<br>• City with less than 100,000 inhabitants<br>• Village
21. Education	• Primary<br>• Secondary vocational<br>• Secondary general<br>• Higher
22. What vaccine did you get?	• Pfizer-BioNTech<br>• AstraZeneca<br>• Moderna<br>• Johnson & Johnson
23. Which dose of vaccine?	• 1st<br>• 2nd<br>• 3rd

Table S2. The reasons for getting vaccinated against COVID-19 at a pharmacy in relation to basic characteristics of participants.

		The pharmacy was the only vaccination site available			Problems with access to my clinic			The opening hours of the pharmacy are fine			The pharmacy is easily accessible			I already have good experiences with the pharmacy service		
	Total	Yes	*p*-value	OR 95%CI	Yes	*p*-value	OR 95%CI	Yes	*p*-value	OR 95%CI	Yes	*p*-value	OR 95%CI	Yes	*p*-value	OR 95%CI
Sex																
Female	226	96 (42.48%)	0.733	-	63 (27.88%)	0.147	-	216 (95.58%)	0.708*	-	215 (95.13%)	0.372*	-	170 (75.22%)	0.855	-
Male	172	76 (44.19 %)			37 (21.51%)			163 (94.77%)			160 (93.02%)			128 (74.42%)		
Age																
G1	171	60 (35.09%)	0.001	ref.	39 (22.81%)	0.634	-	164 (95.91%)	0.789	-	168 (98.25%)	0.008	6.22 (1.72-22.56)	112 (65.5%)	0.0002	ref.
G2	120	51 (42.5%)		1.37 (0.85-2.21)	33 (27.5%)			113 (94.17%)			108 (90%)		ref.	93 (77.5%)		1.81 (1.06-3.09)
G3	107	61 (57.01%)		2.45 (1.49-4.02)	28 (26.17%)			102 (95.63%)			99 (92.52%)		1.37 (0.54-3.5)	93 (86.92%)		3.5 (1.84-6.67)
Place of residence																
city above 100.000	206	80 (39.02%)	0.109	-	49 (23.9%)	0.434	-	197 (96.1%)	0.688	-	192 (93.66%)	0.006	2.46 (0.93-6.54)	144 (70.24%)	0.015	1.04 (0.53-2.05)
city of less than 100.000	143	64 (44.76%)			35 (24.48%)			135 (94.41%)			140 (97.9%)		7.78 (1.93-31.41)	119 (83.22%)		2.19 (1.03-4.63)
rural	49	27 (55.1%)			16 (32.65%)			46 (93.88%)			42 (85.71%)		ref.	34 (69.39%)		ref.
**Education**																
Higher	162	50 (30.86%	0.000	ref.	36 (22.22%)	0.675	-	153 (94.44%)	0.614	-	153 (94.44%)	0.339	-	118 (72.84%)	0.427	-
Secondary general	162	73 (45.06%)		1.84 (1.16-2.89)	43 (26.54%)			155 (95.68%)			152 (93.83%)			121 (74.69%)		
Secondary vocational	50	30 (60%)		3.36 (1.74-6.48)	15 (30%)			49 (98%)			49 (98%)			42 (84%)		
Primary	24	19 (79.17%)		8.51 (3-24.08)	6 (25%)			22 (91.67%)			21 (87.5%)			17 (70.83%)		

Vaccine dose																
1st	30	19 (63.33%)			12 (40%)			26 (86.67%)		ref.	27 (90%)		1.38 (0.34-5.65)	24 (80%)		
2nd	60	27 (45%)	0.058	-	13 (21.67%)	0.135	-	55 (91.67%)	0.017	1.69 (0.42-6.83)	52 (86.67%)	0.009	ref.	48 (80%)	0.444	-
3rd	308	126 (40.91%)			75 (24.35%)			298 (96.75%)		4.58 (1.34-15.63)	296 (96.1%)		3.79 (1.48-9.73)	226 (73.38%)		

age G1: ≤35 years, G2: between 36-50 and G3: ≥ 51; p-value*- test V-square, p-value** test Chi² with Yates correction

Table S3. Method of registration for vaccination in pharmacies in relation to basic characteristics of participants.

		How to register for vaccination at the pharmacy			
	Total	by e-registration. patient gov.pl	24/7 helpline 989	personally or by phone at the pharmacy	*p*-value
Sex					
Female	226	99 (43.81%)	29 (12.83%)	98 (43.36%)	0.283
Male	172	64 (37.21%)	30 (17.44%)	78 (45.35%)	
Age					
G1	171	104 (60.82%)	19 (11.11%)	48 (28.07%)	0.000
G2	120	45 (37.5%)	23 (19.17%)	52 (43.33%)	
G3	107	14 (13.08%)	17 (15.89%)	76 (71.03%)	
Place of residence					
city above 100.000	206	103 (50.24%)	26 (12.68%)	76 (37.07%)	0.002
city of less than 100.000	143	43 (30.07%)	22 (15.38%)	78 (54.55%)	
rural	49	17 (34.69%)	11 (22.45%)	21 (42.86%)	
Education					
Higher	162	90 (55.56%)	16 (9.88%)	56 (34.57%)	0.000
Secondary general	162	60 (37.04%)	29 (17.9%)	73 (45.06%)	
Secondary vocational	50	11 (22%)	5 (10%)	34 (68%)	
Primary	24	2 (8.33%)	9 (37.5%)	13 (54.17%)	
Vaccine dose					
1st	30	13 (43.33%)	7 (23.33%)	10 (33.33%)	0.292
2nd	60	19 (31.67%)	10 (16.67%)	31 (51.67%)	
3rd	308	131 (42.53%)	42 (13.64%)	135 (43.83%)	

age G1: ≤35 years, G2: between 36-50 and G3: ≥ 51

Table S4. Opinions regarding experiences of immunization against COVID-19 at the pharmacy in relation to basic characteristics of participants.

Experience with		the interview prior to vaccination			with the information received prior to vaccination			the technique of vaccination			with the premises organized for vaccination			the post-vaccination care in the pharmacy		
	Total	Yes	*p*-value	OR 95%CI	Yes	*p*-value	OR 95%CI	Yes	*p*-value	OR 95%CI	Yes	*p*-value	OR 95%CI	Yes	*p*-value	OR 95%CI
Sex																
Female	226	208 (92.04%)	0.503	-	216 (95.58%)	0.0007	3.5 (1.63-7.54)	222 (98.23%)	0.054*	3.06 (0.93-10.12)	214 (94.60%)	0.766*	-	204 (90.27%)	0.073	-
Male	172	155 (90.12%)			148 (86.05%)		ref.	163 (94.77%)		ref.	164 (95.35%)			145 (84.30%)		
Age																
G1	171	152 (88.89%)	0.290	-	158 (92.4%)	0.843	-	170 (99.42%)	0.032	10.1 (1.19-85.1)	163 (95.32%)	0.942	-	162 (94.74%)	0.0005	4.5 (2-10.08)
G2	120	113 (94.17%)			109 (90.83%)			114 (95%)		1.13 (0.35-3.61)	114 (95%)			96 (80%)		ref.
G3	107	98 (91.59%)			97 (90.65%)			101 (94.39%)		ref.	101 (94.39%)			91 (85.05%)		1.42 (0.71-2.85)
Place of residence																
city above 100.000	206	181 (88.29%)	0.048	ref.	191 (93.17%)	0.384	-	201 (98.05%)	0.0008	7.01 (1.89-25.92)	195 (95.12%)	0.542	-	189 (92.2%)	0.004	2.88 (1.49-5.54)
city of less than 100.000	143	137 (95.8%)		3.03 (1.2-7.71)	129 (90.21%)			140 (97.9%)		6.51 (1.56-27.14)	137 (95.8%)			115 (80.42%)		ref.
rural	49	44 (89.8%)		1.16 (0.42-3.23)	43 (87.76%)			43 (87.76%)		ref.	45 (91.84%)			44 (89.8%)		2.14 (0.78-5.9)
Education																
Higher	162	153 (94.44%)	0.295	-	152 (93.83%)	0.021	2.39 (1.09-5.22)	157 (96.91%)	0.267	-	154 (95.06%)	0.986	-	149 (91.98%)	0.015	2.39 (1.19-4.81)
Secondary general	162	144 (88.89%)			140 (86.42%)		ref.	154 (95.06%)			154 (95.06%)			134 (82.72%)		ref.

Secondary vocational	50	45 (90%)			49 (98%)		7.7 (1.01-58.64)	50 (100%)			47 (94%)			42 (84%)		1.09 (0.46-2.59)
Primary	24	21 (87.5%)			23 (95.83%)		3.61 (0.46-28.13)	24 (100%)			23 (95.83%)			24 (100%)		-
Vaccine dose																
1st	30	24 (80%)		ref.	28 (93.33%)			25 (83.33%)		ref.	26 (86.67%)		ref.	26 (86.67%)		
2nd	60	48 (80%)	0.0001	ref.	54 (90%)	0.859	-	59 (98.33%)	0.0001	11.8 (1.31-106.21)	52 (86.67%)	0.0002	ref.	55 (91.67%)	0.595	-
3rd	308	291 (94.48%)		4.28 (1.92-9.52)	281 (91.56%)			301 (97.73%)		4.28 (1.54-11.86)	300 (97.4%)		5.77 (1.63-20.45)	268 (87.01%)		

age G1: ≤35 years, G2: between 36-50 and G3: ≥ 51; p-value*- test V-square, p-value** test Chi2 with Yates correction

Table S5. Opinions regarding satisfaction with immunization at the pharmacy in relation to basic characteristics of participants.

Satisfaction with		safe during the vaccination procedure			pharmacists are competent to vaccinate			satisfaction with vaccination in the pharmacy			further vaccinations at the pharmacy		
	Total	Yes	*p*-value	OR 95%CI	Yes	*p*-value	OR 95%CI	Yes	*p*-value	OR 95%CI	Yes	*p*-value	OR 95%CI
Sex													
Female	226	224 (99.12%)	0.451**	-	210 (92.92%)	0.036	2.03 (1.03-3.97)	214 (94.69%)	0.828*	-	202 (89.38%)	0.619	-
Male	172	168 (97.67%)			149 (86.83%)		Ref	163 (94.77%)			151 (87.79%)		
Age													
G1	171	170 (99.42%)			162 (94.74%)		2.77 (1.18-6.5)	163 (95.32%)			156 (91.23%)		
G2	120	117 (97.5%)	0.392	-	104 (86.67%)	0.031	ref.	113 (94.17%)	0.791	-	106 (88.33%)	0.282	-
G3	107	105 (98.13%)			93 (86.92%)		1.02 (0.47-2.21)	100 (93.46%)			91 (85.05%)		
Place of residence													
city above 100.000	206	200 (97.56%)			186 (90.735)			192 (93.66%)			182 (88.78%)		
city of less than 100.000	143	143 (100%)	0.176	-	130 (90.91%)	0.533	-	139 (97.02%)	0.114	-	125 (87.41%)	0.699	-
rural	49	48 (97.96%)			42 (85.71%)			44 (89.8%)			45 (91.84%)		
Education													
Higher	162	162 (100%)		-	148 (91.36%)			151 (93.21%)			152 (93.83%)		3.04 (0.87-10.61)
Secondary general	162	156 (96.3%)	0.031	-	146 (90.12%)	0.869	-	154 (95.06%)	0.823	-	136 (83.95%)	0.034	1.05 (0.33-3.31)
Secondary vocational	50	50 (100%)		-	44 (88%)			48 (96%)			45 (90%)		1.8 (0.44-7.42)

Primary	24	24 (100%)		-	21 (87.5%)			23 (95.83%)			20 (83.33%)	ref.
Vaccine dose												
1st	30	30 (100%)			24 (80%)			28 (93.33%)			25 (83.33%)	
2nd	60	59 (98.33%)	0.779	-	54 (90%)	0.142	-	54 (90%)	0.229	-	50 (83.33%)	0.189	-
3rd	308	303 (98.38%)			281 (91.56%)			294 (95.45%)			278 (90.26%)		

Age G1: ≤35 years, G2: between 36-50 and G3: ≥ 51; p-value*- test V-square, p-value** test Chi^2 with Yates correction

Table S6. Attitude to the expansion of pharmacy services in relation to basic characteristics of participants.

Type of services		Sex		Age			Place of residence			Education				Vaccine dose		
		Female	Male	G1	G2	G3	city above 100.000	city of less than 100.000	Rural	Higher	Secondary general	Secondary vocational	Primary	1st	2nd	3rd
Blood pressure control	Total	210	163	159	119	95	189	138	46	154	152	45	22	29	52	292
	Yes	185 (88.1%)	138 (84.66 %)	131 (82.39%)	110 (92.44%)	82 (86.32%)	167 (88.36%)	118 (85.51%)	38 (82.61%)	141 (91.56%)	124 (81.58%)	39 (86.67%)	19 (86.36%)	23 (79.31%)	45 (86.54%)	255 (87.33%)
	p-value	0.334			0.052			0.528			0.087				0.481	
	OR 95%CI	-			-			-			-				-	
Glucose control	Total	210	162	160	117	95	190	136	46	154	153	44	21	29	52	291
	Yes	182 (86.67%)	140 (86.42 %)	132 (82.5%)	108 (91.45%)	83 (87.37%)	164 (86.32%)	120 (88.24%)	38 (82.61%)	137 (88.96%)	128 (83.66%)	39 (86.64%)	18 (85.71%)	23 (79.31%)	44 (84.62%)	255 (87.63%)
	p-value	0.945			0.094			0.620			0.563				0.414	
	OR 95%CI	-			-			-			-				-	
BMI calculation	Total	209	162	159	117	95	189	135	47	152	151	45	23	29	52	290
	Yes	164 (78.47%)	117 (72.22 %)	114 (71.7%)	97 (82.91%)	70 (73.68%)	140 (74.07%)	104 (77.04%)	37 (78.72%)	123 (80.92%)	104 (68.87%)	33 (73.33%)	21 (91.3%)	19 (65.52%)	36 (69.23%)	226 (77.93%)
	p-value	0.164			0.086			0.727			0.026				0.165	
	OR 95%CI	-			-			-		1.92 (1.13-3.26)	ref.	1.24 (0.59-2.62)	4.74 (1.06-21.07)		-	
Advice about specialized diets	Total	207	162	160	114	95	189	134	46	152	150	45	22	29	53	287
	Yes	149 (71.98%)	127 (78.4 %)	109 (68.13%)	96 (84.21%)	71 (74.74%)	143 (75.66%)	103 (76.87%)	30 (65.22%)	117 (76.97%)	103 (68.67%)	36 (80%)	20 (90.91%)	15 (51.72%)	42 (79.25%)	219 (76.31%)
	p-value	0.159			0.010			0.270			0.070				0.011	
	OR 95%CI	-		ref.	2.5 (1.36-4.56)	1.38 (0.78-2.45)		-			-			ref.	3.56 (1.33-9.55)	3 (1.38-6.54)
Use medical equipment	Total	202	160	155	113	94	182	134	46	147	149	45	21	29	53	280
	Yes	171 (84.65%)	117 (73.13 %)	122 (78.71%)	91 (80.53%)	75 (79.79%)	146 (80.22%)	104 (77.61%)	38 (82.61%)	123 (83.67%)	111 (74.5%)	35 (77.78%)	19 (90.48%)	21 (72.41%)	37 (69.81%)	230 (82.14%)
	p-value	0.007			0.934			0.732			0.138				0.076	
	OR 95%CI	2.03	ref.		-			-			-				-	
Control of quitting smoking	Total	197	157	154	111	89	181	129	44	145	145	44	20	29	51	274
	Yes	113 (57.36%)	93 (59.24 %)	85 (55.19%)	73 (65.77%)	48 (53.93%)	108 (59.67%)	71 (55.05%)	27 (61.36%)	87 (60%)	76 (52.41%)	27 (61.36%)	16 (80%)	13 (44.83%)	29 (56.86%)	164 (59.85%)
	p-value	0.722			0.146			0.647			0.099				0.289	

	OR 95%CI	-			-			-			-				-	
Continuation of ongoing prescriptions by pharmacists	Total	209	159	160	113	95	186	136	46	154	149	45	20	29	54	285
	Yes	188 (89.95%)	142 (89.31%)	143 (89.38%)	101 (89.38%)	86 (90.53%)	175 (94.09%)	117 (86.03%)	38 (82.61%)	138 (89.61%)	133 (89.26%)	41 (91.11%)	18 (90%)	24 (82.76%)	47 (87.04%)	259 (90.88%)
	p-value	0.841			0.951			0.015			0.988				0.309	
	OR 95%CI	-			-		3.35 (1.26-8.89)	1.29 (0.52-3.2)	ref.		-				-	
Monitoring of the therapy with newly drugs	Total	201	179	156	111	91	183	130	45	148	146	44	20	29	53	276
	Yes	149 (74.13%)	112 (71.34%)	113 (72.44%)	84 (75.68%)	64 (70.33%)	140 (76.5%)	90 (69.23%)	31 (68.89%)	116 (78.38%)	95 (65.07%)	33 (75%)	17 (85%)	23 (79.31%)	39 (73.58%)	199 (72.1%)
	p-value	0.555			0.686			0.293			0.039				0.703	
	OR 95%CI	-			-			-		1.94 (1.15-3.27)	ref.	1.61 (0.75-3.45)	3.04 (0.85-10.87)		-	

Age- G1: ≤35 years, G2: between 36-50 and G3: ≥ 51
